# Privacy-Preserving Multi-User Graph Intersection Scheme for Wireless Communications in Cloud-Assisted Internet of Things

**DOI:** 10.3390/s25061892

**Published:** 2025-03-18

**Authors:** Shumei Yang

**Affiliations:** School of Computer Science and Engineering, South China University of Technology, Guangzhou 510006, China; 201610105707@mail.scut.edu.cn

**Keywords:** cloud computing, Internet of Things (IoT), privacy preserving, graph encryption, graph intersection, wireless communications

## Abstract

Cloud-assisted Internet of Things (IoT) has become the core infrastructure of smart society since it solves the computational power, storage, and collaboration bottlenecks of traditional IoT through resource decoupling and capability complementarity. The development of a graph database and cloud-assisted IoT promotes the research of privacy preserving graph computation. We propose a secure graph intersection scheme that supports multi-user intersection queries in cloud-assisted IoT in this article. The existing work on graph encryption for intersection queries is designed for a single user, which will bring high computational and communication costs for data owners, or cause the risk of secret key leaking if directly applied to multi-user scenarios. To solve these problems, we employ the proxy re-encryption (PRE) that transforms the encrypted graph data with a re-encryption key to enable the graph intersection results to be decrypted by an authorized IoT user using their own private key, while data owners only encrypt their graph data on IoT devices once. In our scheme, different IoT users can query for the intersection of graphs flexibly, while data owners do not need to perform encryption operations every time an IoT user makes a query. Theoretical analysis and simulation results demonstrate that the graph intersection scheme in this paper is secure and practical.

## 1. Introduction

Internet of Things (IoT) has promoted the integration of the physical and digital worlds, greatly facilitating our life. However, the exponential growth of IoT devices brings great challenge for localized data processing, which promotes the emergence of cloud-assisted IoT. Cloud-assisted IoT can manage decentralized IoT devices through the cloud platform, providing computational and storage services for resource-limited IoT devices.

Graphs can describe not only diverse types of data but also connections among them. They have significant application value and are applied widely in a range of IoT scenarios such as smart home, smart logistics, smart wearables and so on. Since a large amount of graph data are produced or stored on IoT devices, graph computation tasks such as subgraph matching, shortest path computation, and graph intersection have become important in IoT applications.

The accelerating growth of cloud-assisted IoT promotes data owners with IoT devices to outsource the storage and computational task to the cloud server. However, it causes privacy leakage risk: the original graph data of IoT devices usually contain sensitive information (such as social relationships and medical data), so directly uploading them to the cloud may cause the data to be stolen or misused. To solve this problem, privacy-preserving graph computation in cloud-assisted IoT was born. As one of the most important technologies, graph encryption makes use of cryptography methods to protect the data and relationships of graphs. Much research has been conducted for graph encryption that supports various kinds of graph operations, including shortest distance computation [1,2,3,4,5,6], minimum community search [7], subgraph counting [8], subgraph matching [9,10,11,12,13,14], and so on.

Graph intersection is an operation that searches for common sub structures among graphs of different IoT devices through comparing their vertices and edges. It plays an important role in social network mutual friend recommendation, collaborative data analysis across institutions, etc. Unfortunately, little research aims at the privacy-preserving graph intersection [15,16,17], for which the scheme in [16] computes the graph intersection of two parties, the scheme in [17] enables multiple parties to collaboratively calculate the graph intersection directly among the participants, and the scheme in [15] proposes an outsourced graph intersection scheme of multiple data owners for a single user. Other relevant works study privacy-preserving subgraph matching [9,10,11,12,13,14] and outsourced private set intersection [18,19,20,21,22,23,24]. Some of these prior works only consider a single user; data owners encrypt their graphs using public encryption or symmetric encryption while the query user is directly issued the corresponding secret keys so they can perform decryption to obtain the query results. Other works support multiple users by applying access control over them, where users obtain the decryption key through the access control mechanism.

However, there are some problems in the schemes that IoT users directly obtain the decryption key: first of all, if the IoT user permission changes, data owners have to re-encrypt their graph with new keys, causing large computational and communication costs. Secondly, since a trusted authority or the data owner needs to manage key distribution, graph data can be decrypted once the decryption key leaked.

### 1.1. Contributions

**Figure 1:**
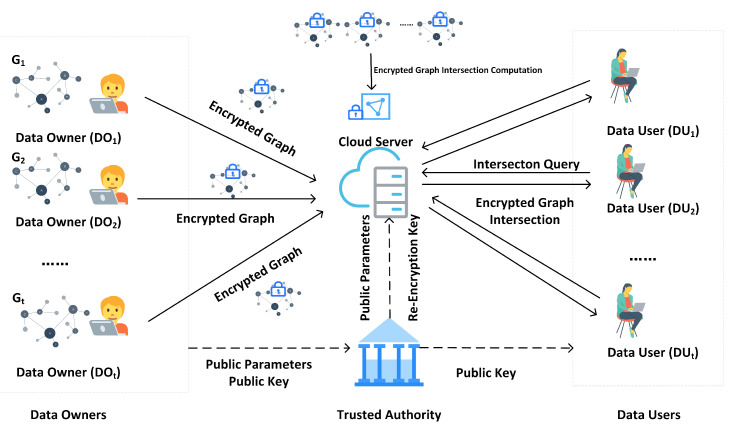
System model.

To enable multiple users to flexibly query graph intersection in a privacy-preserving manner, we present a multi-user graph encryption for intersection queries in the cloud-assisted IoT environment. As is described in Figure 1, the system model consists of four types of entities: a trusted authority (TA), the cloud server, data owners, and data users. The contributions of this paper are summarized as follows.

- We present a construction of privacy-preserving graph intersection computation. In our scheme, a TA initializes the system, and it generates public parameters for the system and re-encryption keys for the cloud server. Each data owner encrypts their graph before uploading it to the cloud server. Every time a data user sends a graph intersection query, the cloud server re-encrypts these encrypted graphs with a re-encryption key from the TA to the data user. After calculating the intersection of all these encrypted graphs, it sends the graph intersection to the query user. Following this procedure, our scheme can support data users to query for the graph intersection of data owners securely and flexibly.
- Our scheme supports multi-user scenarios. It allows multiple users to query for the graph intersection, while data owners only need encrypt their data once. On the one hand, it achieves flexible data sharing; on the other hand, it decreases the processing burden placed on data owners.
- In our scheme, the cloud server transforms the ciphertexts intended for TA to ciphertexts that can be decrypted by the query user by using proxy re-encryption, enabling data users to decrypt the result with their own keys, without exposing sensitive data. It reduces the complexity of key management.
- We present the theoretical analysis from aspects of security and performance. The results from our experiments confirm that the scheme is practical and efficient.

### 1.2. Paper Organization

The related works are summarized in Section 2. We introduce the preliminaries in Section 3. Problem formalization including the system model, threat model, and security goals is presented in Section 4. We give the concrete construction of our scheme in Section 5. The correctness and security analysis are presented in Section 6. In Section 7, we show the performance analysis and experimental evaluation. In the end, we conclude our work in Section 8.

## 2. Related Work

We summarize the related works including graph encryption and outsourced private set intersection. Our scheme represents a specialized form of graph encryption to compute graph intersection, It is an extension of private set intersection, which computes the intersection of sets instead of graphs.

### 2.1. Graph Encryption

Chase and Kamara [25] proposed structured encryption schemes that support several kinds of private queries on encrypted data with complex structures. Among all the graph operations, the shortest distance query is the most fundamental one. Meng et al. [26] proposed GRECS composed of three schemes for different security and efficiency requirements; the schemes realize approximate shortest distance queries. Refs. [4,5,6] support exact shortest distance queries, and refs. [1,2,3] solve the problems in the constrained shortest distance query (CSD). Some other schemes [27,28,29,30] can provide users with the shortest path. Other privacy-preserving graph operations include graph search [31,32], minimum community search [7], graph similarity query [33], subgraph counting [8], and so on. The most related works to ours are subgraph matching [9,10,11,12,13,14] and graph intersection [15]. Cao et al. [14] introduced a system called PPGQ that utilizes the “filtering and verification” principle to filter according to a feature-based index and efficient inner product, where data users decrypt the candidate supergeaphs and verify each candidate. Fan et al. [12] transformed the classic Ullmann’s algorithm as a progression of matrix calculations, which is protected by a cyclic group-based encryption scheme. Zuo et al. [10] designed a privacy-preserving subgraph matching scheme that can protect the privacy of the user’s query subgraph and the original graph. It also achieved data integrity. Ge et al. [11] considered another type of subgraph matching that searches for all graphs exhibiting subgraph isomorphism with the query pattern from large amounts of small graphs, and where the query user is able to directly extract the subgraph. Wang et al. [9] designed OblivGM that supports attributed subgraph matching and can also hide search patterns. Based on OblivGM, they further proposed eGrass [13], which considers secure attributed subgraph matching even if the clouds are malicious. However, the techniques used in these schemes cannot support multiple users flexibly without key management; some of them are designed for a single user, while others enable data users to obtain the decryption key through access control.

### 2.2. Outsourced Private Set Intersection

In outsourced private set intersection (O-PSI), data owners outsource the PSI computation the cloud server. Kerschbaum [18] firstly presented an outsourced PSI scheme on the basis of Bloom filter and HE. Since then, various PSI schemes have been proposed. Ref. [19] extended the scale of PSI protocol to billion-element sets by using a high-efficiency data structure from the Sparsehash library. Abadi et al. [20] presented two delegated private set intersection schemes, of which O-PSI employs additive homomorphic encryption, and EO-PSI makes use of hash tables instead of public key encryption, which provides higher efficiency. Ali et al. [21] designed a protocol in which data owners can define access control policies such that only data owners who satisfy specific attributes can query for PSI results. Since the cloud server can be malicious, the schemes in [22,23,24,34] realized both privacy preservation and verifiability; their PSI computations were combined with the resultant verification mechanisms that enable clients to verify whether the results are correct. Sharma [35] designed a framework named PRISM which is based on secret sharing, where data owners upload their data to non-colluding clouds to perform secure set operations based on secret sharing. All these works support private set intersection among sets, but how to securely compute the intersection of graphs has yet to be studied.

## 3. Preliminaries

We summarize concepts and basic tools of our scheme: to realize secure graph intersection, we employ the proxy re-encryption (PRE) based on bilinear pairings.

### 3.1. Graph Intersection

**Definition** **1.** *(Graph Intersection). Given t graphs $G_{1}=\left(V_{1},E_{1}\right),G_{2}=\left(V_{2},E_{2}\right),\ldots ,G_{t}=\left(V_{t},E_{t}\right)$, the graph intersection is defined as $G=G_{1}\cap G_{2}\cap ...\cap G_{t}=\left(V,E\right)$ satisfying the following:*

- *For each vertex v∈V, $v\in V_{1}$ and $v\in V_{2}$.*
- *For each vertex e∈E, $e\in E_{1}$ and $e\in E_{2}$.*

**Figure 2:**
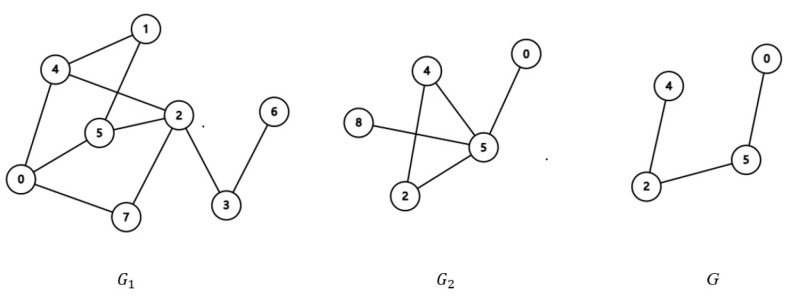
Graph intersection.

*Figure 2 is an example: the intersection of graph $G_{1}$ and $G_{2}$ is G.*

### 3.2. Bilinear Pairings

Bilinear pairings is a map presented as $e:G\times G\to G_{T}$, where G and $G_{T}$ are cyclic groups of prime order *p*, generator $g=\langle G\rangle$. It satisfies the following properties:

- Bilinearity. $e\left(u^{a},v^{b}\right)=e{\left(u,v\right)}^{ab}$, for all u,v∈G and $a,b\in Z_{p}$.
- Non-degeneracy. e(g,g)≠1.
- Computability. e(g,g) can be computed efficiently.

### 3.3. Proxy Re-Encryption

Our graph encryption scheme is based on the proxy re-encryption (PRE) technique. We employ the PRE scheme in the following [36]:

1. PRE.Setup(λ). Given a security parameter λ, TA constructs a bilinear map $e:G\times G\to G_{T}$ where G and $G_{T}$ are groups of prime order *p*, and generator g∈G, return the public parameters $P=\{G,G_{T},e,p,g\}$.
2. PRE.KeyGen(λ). An entity chooses a random $sk\overset{\$}{\leftarrow }Z_{p}^{*}$ as the private key; their public key is $pk\leftarrow g^{sk}$.
3. $PRE.ReKeyGen(sk_{a},pk_{b})$. The re-encryption key $R_{ab}$ can be generated with delegator *a*’s private key $sk_{a}$ and delegatee *b*’s public key as $R_{ab}=pk_{b}^{\frac{1}{sk_{a}}}=g^{\frac{sk_{b}}{sk_{a}}}$
4. $PRE.Enc(m,pk_{a},P)$. To encrypt a message $m\in G_{T}$ under $pk_{a}$: sample $r\overset{\$}{\leftarrow }Z_{p}^{*}$, $C_{1}=pk_{a}^{r}=g^{sk_{a}\cdot r}$, $C_{2}=e{\left(g,g\right)}^{r}\cdot m$. The ciphertext $C=(C_{1},C_{2})$.
5. $PRE.ReEnc(C,R_{ab})$. Given the re-encryption key $R_{ab}$, ciphertext $C=(C_{1},C_{2})$ can be re-encrypted as follows: sample $r^{\prime }\overset{\$}{\leftarrow }Z_{p}^{*}$, $C_{3}={R_{ab}}^{\frac{1}{r^{\prime }}}=pk_{b}^{\frac{1}{sk_{a}\cdot r^{\prime }}}=g^{\frac{sk_{b}}{sk_{a}\cdot r^{\prime }}}$, $C_{4}={C_{1}}^{r^{\prime }}=pk_{i}^{r\cdot r^{\prime }}=g^{sk_{a}\cdot r\cdot r^{\prime }}$. The re-encrypted ciphertext $C^{\prime }=\left(C_{2},C_{3},C_{4}\right)$.
6. $PRE.Dec(C^{\prime },sk_{b})$. Entity *b* can decrypt $C^{\prime }=\left(C_{2},C_{3},C_{4}\right)$ using their private key $sk_{b}$: $m=\frac{C_{2}}{e{\left(C_{3},C_{4}\right)}^{\frac{1}{sk_{b}}}}$

## 4. Problem Formalization

### 4.1. System Model

There are four types of roles in the system—data owners, data users, cloud server, and a trusted authority, as shown in Figure 1:

- Cloud Server. The cloud server possesses strong storage and computational capabilities: it receives and stores encrypted graph data uploaded by data owners, performs graph re-encryption and intersection operations, and finally it provides the results to the data user.
- Data Owner $DO_{i}$. Each data owner has a graph $G_{i}=\left(V_{i},E_{i}\right)$ that participates in graph intersection computation. To ensure the confidentiality of the graph $G_{i}$, they will encrypt $G_{i}$ before uploading it to the cloud server.
- Data User. A data user may query for the intersection of graphs from $DO_{1},DO_{2},\ldots ,DO_{t}$. In order to save storage and computational costs, they outsource the computational task to the cloud server, and finally obtains the encrypted result from the cloud server and decrypts it.
- Trusted Authority. As a trusted third party, the TA initializes the system with a security parameter λ, generates a set of public parameters P. It is also responsible for generating re-encryption keys that enable the cloud server to convert the ciphertexts for them to be decryptable by the data user.

### 4.2. Threat Model

We consider TA and data owners to be trustworthy, data owners will honestly model their graph data and encrypt them. The cloud server is considered semi-honest, implying that it executes our protocol honestly, but it may try to infer sensitive information during computation such as the original and intersection graph, through methods such as statistics and analysis.

### 4.3. Security Goals

- Graph data confidentiality. Any information about original graphs except for information in leakage functions should not be obtainable by the cloud server or other adversaries; only the part in the intersection with graphs from other data owners can be learned by an authorized data user.
- Query result confidentiality. The graph intersection results in a ciphertext form that can only be decrypted by an authorized data user with their own key. It remains confidential from the cloud server, data owners, other data users, and adversaries.

### 4.4. Security Definition

We adopt the adaptive chosen query attack (CQA2) security definition in the graph intersection scheme, which is defined as follows:

**Definition** **2.** *(CQA2-Security). Let Π=(Setup,KeyGen,ReKeyGen,GraphIntersection,Dec) be our private graph intersection scheme, and let $L_{1}$ and $L_{2}$ be leakage functions. A denotes the adversary, and S denotes the simulator. Supposing λ is the security parameter, the experiments in the ideal world and real world are defined as follows:*

- *${\mathrm{Real}}_{A}\left(\lambda \right)$: A outputs graphs $G_{1},G_{2},\ldots ,G_{n}$. The experiment generates a pair of keys (pk,sk) by KeyGen and generates the re-encryption key $R_{TA\to DU}$ by ReKeyGen. Then, A makes queries for intersections of randomly chosen t graphs $G_{1},G_{2},\ldots ,G_{t}$, for each query, and the experiment computes computes $C_{i}\leftarrow Enc\left(G_{i}\right)$ and sends the encrypted graphs $C_{1},C_{2},\ldots ,C_{t}$ to A. It then compute the encrypted intersection graph $\tilde{C}\leftarrow$GraphIntersection$\left(P,R_{TA\to DU},C_{1},\ldots ,C_{t}\right)$ and gives it to A. At the end of the experiment, A outputs a bit $b\in \left\{0,1\right\}$ as the experiment result.*
- *${\mathrm{Ideal}}_{A,S}\left(\lambda \right)$: A outputs graphs $G_{1},G_{2},\ldots ,G_{n}$. Then, A makes queries for intersections of randomly chosen t graphs $G_{1},G_{2},\ldots ,G_{t}$. Based on leakage functions $L_{1}$ and $L_{2}$, S produces encrypted graphs $C_{1}^{*},C_{2}^{*},\ldots ,C_{t}^{*}$ and sends them to A, then S simulates and sends the query results ${\tilde{C}}^{*}$ to A. At the end of the experiment, A outputs a bit $b\in \left\{0,1\right\}$ as the experiment result.*

*We say the graph encryption scheme* Π *is $\left(L_{1},L_{2}\right)$-secure against the adaptive chosen query attack if for any probability polynomial time (PPT) adversary A, there exists a PPT simulator S that*

(1) $$\left|Pr\left[{\mathrm{Real}}_{A}\left(\lambda \right)=1\right]-Pr\left[{\mathrm{Ideal}}_{A,S}\left(\lambda \right)=1\right]\right|\leq negl\left(\lambda \right)$$

*where negl(λ) denotes a negligible function.*

## 5. Construction of Our Scheme

We present our scheme for secure graph intersection computation, including the construction overview and concrete construction.

### 5.1. Construction Overview

Our scheme consists of the following six algorithms:

1. Setup(λ)→P. The procedure setup is executed by the TA, the trusted third party, taking a secure parameter λ as input and producing a set of public parameters P as output.
2. KeyGen(λ,P)→(pk,sk). Upon input of a security parameter λ and public parameter P, we use this algorithm to generate a pair of public–private keys (pk,sk).
3. $ReKeyGen\left(P,sk,pk_{DU}\right)\to R_{TA\to DU}$. Given the public parameter P, TA’s private key sk, and the public key $pk_{DU}$ of the data user DU, the algorithm outputs a re-encryption key $R_{TA\to DU}$ that allows ciphertexts encrypted by data owners to be transformed into ciphertexts intended for the data user DU.
4. $Enc\left(P,pk,G_{i}\right)\to C_{i}$. It is the graph encryption algorithm executed by data owner $DO_{i}$; taking the graph $G_{i}$, public key of the TA, and P as inputs, it outputs the encrypted graph $C_{i}$.
5. $GraphIntersection\left(P,R_{TA\to DU},C_{1},\ldots ,C_{t}\right)\to \tilde{C}$. This algorithm takes public parameter P, the re-encryption key $R_{TA\to DU}$, and ciphertexts $C_{1},C_{2},\ldots ,C_{t}$ uploaded by the data owner $DO_{1},DO_{2},\ldots ,DO_{t}$, and the cloud server performs re-encryption on $C_{1},C_{2},\ldots ,C_{t}$, resulting in new ciphertexts ${\tilde{C}}_{1},\ldots ,{\tilde{C}}_{t}$. The cloud server calculates the graph intersections and outputs the encrypted result $\tilde{C}$.
6. $Dec(P,\tilde{C},sk_{DU})\to G$.Taking the inputs of private key $sk_{DU}$ and re-encrypted ciphertext $\tilde{C}$, this algorithm returns the subgraph *G*.

### 5.2. Concrete Construction

The details of our secure graph intersection scheme is described in this section. We summarize the notations in our construction in Table 1.

#### 5.2.1. Setup

Given the security parameter λ, TA completes the setup phase and generates the following public parameters: a bilinear map $e:G\times G\to G_{T}$, where G and $G_{T}$ are groups of prime order *p*, and generator g∈G, a collision-resistant hash function $H:{\left\{0,1\right\}}^{*}\to Z_{p}$. The public parameters $P=\{G,G_{T},p,g,H\}$.

#### 5.2.2. KeyGen

TA chooses the private key sk randomly from $Z_{p}^{*}$, and the public key is $pk\leftarrow g^{sk}$. Similarly, data user $DU_{i}$ also generates their own public–private key pair $\left(pk_{DU},sk_{DU}\right)$, where $pk_{DU}=g^{sk_{DU}}$.

#### 5.2.3. ReKeyGen

When user DU initiates a query request, TA generates the re-encryption key for DU: $R_{TA\to DU}={\left(pk_{DU}\right)}^{\frac{1}{sk}}$.

#### 5.2.4. Enc

Data owner $DO_{i}$ models their graph as $G_{i}=\left(V_{i},E_{i}\right)$, $V_{i}=\left\{v_{1}^{i},v_{2}^{i},\ldots v_{n_{i}}^{i}\right\}$ is the vertex set, and each $v_{j}^{i}$ represents the unique ID value of a vertex. $E_{i}$ is the adjacency matrix, which can be represented as

$$E_{i}=\left(\begin{array}{ccc} e_{11}^{i} & \ldots & e_{1n_{i}}^{i}\\ \ldots & & \ldots \\ e_{n_{i}1}^{i} & \ldots & e_{n_{i}n_{i}}^{i} \end{array}\right)$$

where each element $e_{kl}^{i}\in \left\{\theta ,1\right\}$ (θ is a random number that θ≠0), $e_{kl}^{i}=1$ indicates that there is an edge connecting nodes $v_{k}^{i}$ and $v_{l}^{i}$, while $e_{kl}^{i}=\theta$ indicates that there is no edge between the two nodes.

To ensure the confidentiality of the graph, $DO_{i}$ performs the following operations on $G_{i}=\left(V_{i},E_{i}\right)$ as shown in Algorithm 1.

- Vertices Hashing. $DO_{i}$ performs a hash computation on the vertices set to obtain the corresponding hashed set $H_{i}=\left\{h_{1}^{i},h_{2}^{i},\ldots ,h_{n_{i}}^{i}\right\}$.
- Graph Encryption. $DO_{i}$ encrypts $G_{i}=\left(V_{i},E_{i}\right)$ using proxy re-encryption (PRE). To elaborate in detail, given $V_{i}=\left\{v_{1}^{i},v_{2}^{i},\ldots v_{n_{i}}^{i}\right\}$, adjacency matrix $E_{i}$, pk. For each element $v_{j}^{i}\in V_{i}$, choose $r_{j}^{i}$ randomly from $Z_{p}^{*}$, compute $c_{j}^{i}=\left(c_{1j}^{i},c_{2j}^{i}\right)$, where $c_{1j}^{i}=pk^{r_{j}^{i}},c_{2j}^{i}=e{\left(g,g\right)}^{r_{j}^{i}}\cdot v_{j}^{i}$. For each element $e_{kl}^{i}\in E_{i}$, choose $r_{kl}^{i}$ randomly from $Z_{p}^{*}$, and compute $c_{kl}^{i}=\left(c_{1kl}^{i},c_{2kl}^{i}\right)$ where $c_{1kl}^{i}=pk^{r_{kl}^{i}},c_{2kl}^{i}=e{\left(g,g\right)}^{r_{kl}^{i}}\cdot e_{kl}^{i}$. The encrypted graph is $C^{i}=\left(C_{v}^{i},C_{e}^{i}\right)$, where the encrypted vertices set $C_{v}^{i}=\left\{c_{1}^{i},c_{2}^{i},\ldots ,c_{n_{i}}^{i}\right\}$, and the encrypted adjacency matrix
  $$C_{e}^{i}=\left(\begin{array}{ccc} c_{11}^{i} & \ldots & c_{1n_{i}}^{i}\\ \ldots & & \ldots \\ c_{n_{i}1}^{i} & \ldots & c_{n_{i}n_{i}}^{i} \end{array}\right)$$

**Algorithm 1** Enc.**Input**: public parameter P, $DO_{i}$’s graph $G_{i}=\left(V_{i},E_{i}\right)$, TA’s public key pk. **Output:** encrypted graph $C^{i}$.
1:**for** each element $v_{j}^{i}\in V_{i}$ **do**2:   $h_{j}^{i}\leftarrow H\left(v_{j}^{i}\right)$.3:   $c_{j}^{i}\leftarrow PRE.Enc\left(v_{j}^{i},pk,P\right)$.4:**end for**5:$H_{i}\leftarrow \left\{h_{1}^{i},h_{2}^{i},\ldots ,h_{n_{i}}^{i}\right\}$.6:$C_{v}^{i}\leftarrow \left\{c_{1}^{i},c_{2}^{i},\ldots ,c_{n_{i}}^{i}\right\}$.7:**for** each element $e_{kl}^{i}\in E_{i}$ **do**8:   $c_{kl}^{i}\leftarrow PRE.Enc\left(e_{kl}^{i},pk,P\right)$.9:**end for**10:Set$$C_{e}^{i}=\left(\begin{array}{ccc} c_{11}^{i} & \ldots & c_{1n_{i}}^{i}\\ \ldots & & \ldots \\ c_{n_{i}1}^{i} & \ldots & c_{n_{i}n_{i}}^{i} \end{array}\right)$$11:**return**$\;H_{i},C^{i}=\left(C_{v}^{i},C_{e}^{i}\right)$.


#### 5.2.5. GraphIntersection

The GraphIntersection in Algorithm 2 works as follows:
**Algorithm 2** GraphIntersection.**Input:** public parameter P, the re-encryption key $R_{TA\to DU}$, $C^{i}=\left(C_{v}^{i},C_{e}^{i}\right)$, $H_{i}$, i=1,2,…,t.
**Output:** 
$\tilde{C}$.
1:$H_{I}=\left\{h_{1},h_{2},\ldots ,h_{s}\right\}\leftarrow Intersection\left(H_{1},H_{2},\ldots ,H_{t}\right)$.2:Obtain the encrypted vertices set $C_{I}=\left\{c_{I(j)},j=1,\ldots ,s\right\}$ according to $H_{I}$.3:**for** each element $c_{I(j)}\in C_{I}$ **do**4:   ${\tilde{c}}_{I_{(}j)}\leftarrow PRE.ReEnc\left(c_{I(j)},R_{TA\to DU}\right)$.5:**end for**6:The re-encrypted node set ${\tilde{C}}_{I}\leftarrow \left\{{\tilde{c}}_{I(j)},j=1,\ldots ,s\right\}$.7:**for** each encrypted matrix $C_{e}^{i}$, i=1,2,…,t **do**8:   Choose the elements $c_{I_{u}\left(i\right)I_{v}\left(i\right)}^{i}$ in the encrypted matrix $C_{e}^{i}$ where u,v∈{1,2,..,s} and constructs the submatrix $S_{e}^{i}$9:   **for** each element $c_{I_{u}\left(i\right)I_{v}\left(i\right)}^{i}\in S_{e}^{i}$ **do**10:     ${\tilde{c}}_{I_{u}\left(i\right)I_{v}\left(i\right)}^{i}\leftarrow PRE.ReEnc\left(c_{I_{u}\left(i\right)I_{v}\left(i\right)}^{i},R_{TA\to DU}\right)$.11:   **end for**12:   The re-encrypted matrix ${\tilde{S}}_{e}^{i}$13:**end for**14:Compute the element-wise product of re-encrypted matrices ${\tilde{C}}_{e}\leftarrow {\tilde{S}}_{e}^{1}\circ \ldots \circ {\tilde{S}}_{e}^{t}$.15:**return** $\tilde{C}=\left({\tilde{C}}_{I},{\tilde{C}}_{e}\right)$.

- Vertices Re-encryption. Given the hash sets $H_{1},H_{2},\ldots ,H_{t}$, the cloud server computes their intersection. Denote the intersection by $H_{I}=H_{1}\cap H_{2}\cap \ldots \cap H_{t}=\left\{h_{1},h_{2},\ldots ,h_{s}\right\}$ and the intersection of graph nodes as $V_{I}=V_{1}\cap V_{2}\cap \ldots \cap V_{t}=v_{1},v_{2},\ldots ,v_{s}$. The graph node corresponding to element $h_{j}$ actually has different ordinality in the original graphs. We denote the ordinality of the node of the original graph $G_{i}$ corresponding to $h_{j}$ by $I_{i}\left(j\right):\left[1..s\right]\to \left[1..n_{s}\right]$. There are *t* encrypted node sets, and since the *t* encrypted node sets can be decrypted to the same node intersection set, we only need to choose a random one to re-encrypt it, which can be written as $C_{I}=\left\{c_{I(j)},j=1,\ldots ,s\right\}$. For each $c_{I(j)}\in C_{I}$, we re-encrypt it using proxy re-encryption (PRE). Specifically, for $c_{I(j)}=\left(c_{1I(j)},c_{2I(j)}\right)=\left(pk^{r_{I(j)}},e{\left(g,g\right)}^{r_{I(j)}}\cdot v_{I(j)}\right)$, choose $t_{I(j)}$ randomly from $Z_{p}^{*}$, and compute $c_{3I(j)}=R_{TA\to DU}^{\frac{1}{t_{I(j)}}},c_{4I(j)}=c_{1I(j)}^{t_{I(j)}}$, with the re-encrypted ${\tilde{c}}_{I(j)}=\left(c_{3I(j)},c_{4I(j)},c_{2I(j)}\right)$. The cloud server can precompute $e(c_{3I(j)},c_{4I(j)})$ for every node for the decryption phase. Finally, the cloud server obtains the re-encrypted vertices sets ${\tilde{C}}_{I}\leftarrow \left\{{\tilde{c}}_{I(j)},j=1,\ldots ,s\right\}$.
- Matrices Re-encryption. The cloud server continues to calculate the re-encrypted adjacency matrix. It chooses the elements $c_{I_{u}\left(i\right)I_{v}\left(i\right)}^{i}$ in the encrypted matrix $C_{e}^{i}$ where u,v∈{1,2,..,s} and constructs the submatrix
  $$S_{e}^{i}=\left(\begin{array}{ccc} c_{I_{1}\left(i\right)I_{1}\left(i\right)}^{i} & \ldots & c_{I_{1}\left(i\right)I_{s}\left(i\right)}^{i}\\ \ldots & & \ldots \\ c_{I_{s}\left(i\right)I_{1}\left(i\right)}^{i} & \ldots & c_{I_{s}\left(i\right)I_{s}\left(i\right)}^{i} \end{array}\right)$$
  For each $c_{I_{u}\left(i\right)I_{v}\left(i\right)}^{i}\in S_{e}^{i}$, we re-encrypt it using proxy re-encryption (PRE). Specifically, for $c_{I_{u}\left(i\right)I_{v}\left(i\right)}^{i}=\left(c_{1I_{u}\left(i\right)I_{v}\left(i\right)},c_{2I_{u}\left(i\right)I_{v}\left(i\right)}\right)=\left(pk^{r_{I_{u}\left(i\right)I_{v}\left(i\right)}^{i}},e{\left(g,g\right)}^{r_{I_{u}\left(i\right)I_{v}\left(i\right)}^{i}}\cdot e_{I_{u}\left(i\right)I_{v}\left(i\right)}^{i}\right)$, we choose $t_{I_{u}\left(i\right)I_{v}\left(i\right)}^{i}$ randomly from $Z_{p}^{*}$, and compute $c_{3I_{u}\left(i\right)I_{v}\left(i\right)}^{i}=R_{TA\to DU}^{\frac{1}{t_{I_{u}\left(i\right)I_{v}\left(i\right)}^{i}}},c_{4I_{u}\left(i\right)I_{v}\left(i\right)}^{i}=c_{1I_{u}\left(i\right)I_{v}\left(i\right)}^{t_{I_{u}\left(i\right)I_{v}\left(i\right)}^{i}}$, with the re-encrypted ${\tilde{c}}_{I_{u}\left(i\right)I_{v}\left(i\right)}^{i}=\left(c_{3I_{u}\left(i\right)I_{v}\left(i\right)}^{i},c_{4I_{u}\left(i\right)I_{v}\left(i\right)}^{i},c_{2I_{u}\left(i\right)I_{v}\left(i\right)}^{i}\right)$. The cloud server can also precompute $e(c_{3I_{u}\left(i\right)I_{v}\left(i\right)}^{i},c_{4I_{u}\left(i\right)I_{v}\left(i\right)}^{i})$ for every element for the decryption phase. Finally, the cloud server obtains the re-encrypted matrix
  $${\tilde{S}}_{e}^{i}=\left(\begin{array}{ccc} c_{2I_{1}\left(i\right)I_{1}\left(i\right)}^{i} & \ldots & c_{2I_{1}\left(i\right)I_{s}\left(i\right)}^{i}\\ \ldots & & \ldots \\ c_{2I_{s}\left(i\right)I_{1}\left(i\right)}^{i} & \ldots & c_{2I_{s}\left(i\right)I_{s}\left(i\right)}^{i} \end{array}\right)$$
- Graph Intersection Computation. The cloud server then computes
  (2) $$\begin{array}{cc} \; & {\tilde{C}}_{e}={\tilde{S}}_{e}^{1}\circ ...\circ {\tilde{S}}_{e}^{t}=\left(\begin{array}{ccc} \prod_{i=1}^{t}c_{2I_{1}\left(i\right)I_{1}\left(i\right)}^{i} & ... & \prod_{i=1}^{t}c_{2I_{1}\left(i\right)I_{s}\left(i\right)}^{i}\\ ... & \; & ...\\ \prod_{i=1}^{t}c_{2I_{s}\left(i\right)I_{1}\left(i\right)}^{i} & ... & \prod_{i=1}^{t}c_{2I_{s}\left(i\right)I_{s}\left(i\right)}^{i} \end{array}\right) \end{array}$$

At the end, the calculated result $\tilde{C}=\left({\tilde{C}}_{I},{\tilde{C}}_{e}\right)$ is sent to the querying user DU.

#### 5.2.6. Dec

The Dec shown in Algorithm 3 works as follows:

- Vertices Decryption. According to the graph re-encryption in Algorithm 2, the re-encrypted node set corresponding to the intersection set $V_{I}$ is ${\tilde{C}}_{I}=\left\{{\tilde{c}}_{I(j)},j=1,\cdots ,s\right\}$, where ${\tilde{c}}_{I(j)}=\left(c_{3I(j)},c_{4I(j)},c_{2I(j)}\right)=\left(R_{TA\to DU}^{\frac{1}{t_{I(j)}}},pk^{r\cdot t_{I(j)}},e{\left(g,g\right)}^{r_{j}}\cdot v_{j}\right)=\left(g^{\frac{sk_{DU}}{sk\cdot t}},g^{sk\cdot r\cdot t_{I(j)}},e{\left(g,g\right)}^{r_{j}}\cdot v_{j}\right)$. It can be obtained that $v_{j}=\frac{c_{2I(j)}}{e{\left(c_{3I(j)},c_{4I(j)}\right)}^{\frac{1}{sk_{DU}}}}$. Finally, we obtain the set of intersections of vertices $V=\{v_{j},j=1,\cdots ,s\}$.
- Matrix Decryption. The edges set intersection ${\tilde{C}}_{e}$ can be decrypted with $sk_{DU}$ as follows:
  (3) $$\begin{array}{cc} \; & E=PRE.Dec_{sk_{DU}}\left({\tilde{C}}_{e},sk_{DU}\right)\\ \; & =PRE.Dec_{sk_{DU}}\left(\begin{array}{ccc} \prod_{i=1}^{t}c_{2I_{1}\left(i\right)I_{1}\left(i\right)}^{i} & \ldots & \prod_{i=1}^{t}c_{2I_{1}\left(i\right)I_{s}\left(i\right)}^{i}\\ \ldots & \; & \ldots \\ \prod_{i=1}^{t}c_{2I_{s}\left(i\right)I_{1}\left(i\right)}^{i} & ... & \prod_{i=1}^{t}c_{2I_{s}\left(i\right)I_{s}\left(i\right)}^{i} \end{array}\right)\\ \; & =\left(\begin{array}{ccc} \prod_{i=1}^{t}e_{I_{1}\left(i\right)I_{1}\left(i\right)}^{i} & ... & \prod_{i=1}^{t}e_{I_{1}\left(i\right)I_{s}\left(i\right)}^{i}\\ ... & \; & ...\\ \prod_{i=1}^{t}e_{I_{s}\left(i\right)I_{1}\left(i\right)}^{i} & ... & \prod_{i=1}^{t}e_{I_{s}\left(i\right)I_{s}\left(i\right)}^{i} \end{array}\right) \end{array}$$

Finally, the data user DU recovers the intersection of the graphs *G* by using *V* and *E*.
**Algorithm 3** Dec.**Input:** $\tilde{C}=\left({\tilde{C}}_{I},{\tilde{C}}_{e}\right)$, P, $sk_{DU}$. **Output:** *G*.
1:**for** ${\tilde{c}}_{I(j)}\in {\tilde{C}}_{I}$ **do**2:   Decrypt $v_{j}=PRE.Dec\left({\tilde{c}}_{I(j)},sk_{DU}\right)$.3:**end for**4:$V\leftarrow \{v_{1},v_{2},\ldots ,v_{s}\}$.5:$E\leftarrow PRE.Dec_{sk_{DU}}\left({\tilde{C}}_{e},sk_{DU}\right)$6:**return** G=(V,E)

## 6. Correctness and Security Analysis

### 6.1. Correctness Analysis

**Theorem** **1.** *If all follow the algorithms within the scheme, then the query user can obtain the correct graph intersection result.*

**Proof.**  We prove that the query user can obtain $G=\left(V,E\right)=G_{1}\cap G_{2}\cap ...\cap G_{t}$.

When calculating graph intersection, the cloud server first acquires a set of common hash values $H_{I}=\left\{h_{1},h_{2},...,h_{s}\right\}$. Since each value in the hash set $H_{i}$ corresponds uniquely to an element in the ciphertext set $C_{v}^{i}$, the encrypted node subset can be correctly extracted from $C_{v}^{i}$ as $C_{I}=\left\{c_{I(j)},j=1,...,s\right\}$. The cloud server then re-encrypts it to obtain the re-encrypted node set ${\tilde{C}}_{I}\leftarrow \left\{{\tilde{c}}_{I(j)},j=1,...,s\right\}$, which can be decrypted to obtain $V_{I}=V_{1}\cap V_{2}\cap ...\cap V_{t}=\left\{v_{j},j=1,\cdots ,s\right\}$, to be specific, given ${\tilde{c}}_{I(j)}=\left(c_{3I(j)},c_{4I(j)},c_{2I(j)}\right)$ and $sk_{DU}$, $v_{j}=\frac{c_{2I(j)}}{e{\left(c_{3I(j)},c_{4I(j)}\right)}^{\frac{1}{sk_{DU}}}}$.

The cloud server constructs the submatrices, re-encrypts the elements in the matrices and performs the Hadamard product on these matrices to obtain

(4) $$\begin{array}{cc} \; & {\tilde{C}}_{e}={\tilde{S}}_{e}^{1}\circ \ldots \circ {\tilde{S}}_{e}^{t}=\left(\begin{array}{ccc} \prod_{i=1}^{t}c_{2I_{1}\left(i\right)I_{1}\left(i\right)}^{i} & ... & \prod_{i=1}^{t}c_{2I_{1}\left(i\right)I_{s}\left(i\right)}^{i}\\ ... & \; & ...\\ \prod_{i=1}^{t}c_{2I_{s}\left(i\right)I_{1}\left(i\right)}^{i} & ... & \prod_{i=1}^{t}c_{2I_{s}\left(i\right)I_{s}\left(i\right)}^{i} \end{array}\right) \end{array}$$

The data user decrypts to obtain the intersection matrix with their private key $sk_{DU}$

(5) $$\begin{array}{cc} \; & E=\left(\begin{array}{ccc} \prod_{i=1}^{t}e_{I_{1}\left(i\right)I_{1}\left(i\right)}^{i} & ... & \prod_{i=1}^{t}e_{I_{1}\left(i\right)I_{s}\left(i\right)}^{i}\\ ... & \; & ...\\ \prod_{i=1}^{t}e_{I_{s}\left(i\right)I_{1}\left(i\right)}^{i} & ... & \prod_{i=1}^{t}e_{I_{s}\left(i\right)I_{s}\left(i\right)}^{i} \end{array}\right)\\ \; & =\left(\begin{array}{ccc} \frac{\prod_{i=1}^{t}e_{I_{1}\left(i\right)I_{1}\left(i\right)}^{i}\cdot e{\left(g,g\right)}^{\sum r_{I_{1}\left(i\right)I_{1}\left(i\right)}}}{e{\left(g,g\right)}^{\sum r_{I_{1}\left(i\right)I_{1}\left(i\right)}}} & ... & \frac{\prod_{i=1}^{t}e_{I_{1}\left(i\right)I_{s}\left(i\right)}^{i}\cdot e{\left(g,g\right)}^{\sum r_{I_{1}\left(i\right)I_{s}\left(i\right)}}}{e{\left(g,g\right)}^{\sum r_{I_{1}\left(i\right)I_{1s}}}}\\ ... & \; & ...\\ \frac{\prod_{i=1}^{t}e_{I_{s}\left(i\right)I_{1}\left(i\right)}^{i}\cdot e{\left(g,g\right)}^{\sum r_{I_{s}\left(i\right)I_{1}\left(i\right)}}}{e{\left(g,g\right)}^{\sum r_{I_{s}\left(i\right)I_{1}\left(i\right)}}} & ... & \frac{\prod_{i=1}^{t}e_{I_{s}\left(i\right)I_{1s}}^{i}\cdot e{\left(g,g\right)}^{\sum r_{I_{s}\left(i\right)I_{s}\left(i\right)}}}{e{\left(g,g\right)}^{\sum r_{I_{s}\left(i\right)I_{s}\left(i\right)}}} \end{array}\right)\\ \; & =\left(\begin{array}{ccc} \frac{\prod_{i=1}^{t}c_{2I_{1}\left(i\right)I_{1}\left(i\right)}^{i}}{\prod e{\left(c_{3I_{1}\left(i\right)I_{1}\left(i\right)}^{i},c_{4I_{1}\left(i\right)I_{1}\left(i\right)}^{i}\right)}^{1/sk_{DU}}} & ... & \frac{\prod_{i=1}^{t}c_{2I_{1}\left(i\right)I_{s}\left(i\right)}^{i}}{\prod e{\left(c_{3I_{1}\left(i\right)I_{s}\left(i\right)}^{i},c_{4I_{1}\left(i\right)I_{s}\left(i\right)}^{i}\right)}^{1/sk_{DU}}}\\ ... & \; & ...\\ \frac{\prod_{i=1}^{t}c_{2I_{s}\left(i\right)I_{1}\left(i\right)}^{i}}{\prod e{\left(c_{3I_{s}\left(i\right)I_{1}\left(i\right)}^{i},c_{4I_{s}\left(i\right)I_{1}\left(i\right)}^{i}\right)}^{1/sk_{DU}}} & ... & \frac{\prod_{i=1}^{t}c_{2I_{s}\left(i\right)I_{s}\left(i\right)}^{i}}{\prod e{\left(c_{3I_{s}\left(i\right)I_{s}\left(i\right)}^{i},c_{4I_{s}\left(i\right)I_{s}\left(i\right)}^{i}\right)}^{1/sk_{DU}}} \end{array}\right) \end{array}$$

□

### 6.2. Security Analysis

This section conducts a comprehensive security evaluation of the proposed scheme. Our analysis unfolds in two key phases: initially formalizing the leakage functions, followed by a rigorous proof that the scheme is CQA-2 secure.

- Leakage function $L_{1}$: Given a query $q=(G_{1},G_{2},...,G_{t})$, where $G_{i}=\left(V_{i},E_{i}\right)$, the leakage function $L_{1}$ reveals the information inferred from encrypted graphs $C_{1}^{*},C_{2}^{*},\ldots ,C_{t}^{*}$ and their encrypted intersection graph ${\tilde{C}}^{*}$, including the vertex count of each individual graph and the vertex count of the graph intersection. Thus, $L_{1}\left(q\right)=\left(Num_{1},Num_{2}\right)$ where $Num_{1},\;Num_{2}$ are formally described as follows:
  - $Num_{1}$. $Num_{1}$ is is a *t*-sized array, where $Num_{1}\left[i\right]=\left|V_{i}\right|$ for i=1,2,…,t.
  - $Num_{2}$. $Num_{2}$ signifies the total vertices in the graph intersection which denoted as $|V_{I}|$.
- Leakage function $L_{2}$: The leakage function $L_{2}$ reveals information during multiple queries including query pattern leakage, which reveals whether a particular query has been issued previously, and intersection pattern leakage, which indicates the number of common vertices shared among different queries. Let $q=\left\{q_{1},q_{2},...,q_{m}\right\}$ be a sequence of graph intersection queries, where $q_{i}$ corresponds to a collection of graphs $\left(G_{1}^{i},G_{2}^{i},...,G_{t}^{i}\right)$. They are formally stated as follows.

**Definition** **3.** *(Query pattern leakage). The query pattern leakage function $L_{QP}\left(q\right)$ is modeled as a m×m matrix, where each entry (i,j) signifies whether $q_{i}$ and $q_{j}$ are identical. We denote each entry (i,j) as $Sim\left(q_{i},q_{j}\right)=\left(G_{1}^{i}=G_{1}^{j},G_{2}^{i}=G_{2}^{j},...,G_{t}^{i}=G_{t}^{j}\right)$.*

**Definition** **4.** *(Intersection pattern leakage). The intersection pattern leakage function $L_{IP}\left(q\right)$ is represented as a m×m matrix, where each entry (i,j) contains common hashes between graph intersections corresponding to queries $q_{i}$ and $q_{j}$, denoted as $Com(q_{i},q_{j})$. Since the hash function is deterministic, hash values have one-to-one correspondence with vertices, and $Com(q_{i},q_{j})$ indicates the common vertices between $q_{i}$ and $q_{j}$ without leaking their identities.*

Thus, the leakage function $L_{2}=\left(L_{QP}\left(q\right),L_{IP}\left(q\right)\right)$.

**Theorem** **2.** *If H is a secure hash function and PRE is a secure proxy re-encryption algorithm, then our graph encryption scheme* Π* is $\left(L_{1},L_{2}\right)$-secure against an adaptive chosen query attack.*

**Proof.**  To demonstrate the security of our scheme, we construct a simulator S. Based on $L_{1}$, $L_{2}$, S generates counterfeit encrypted graphs $C_{1}^{*},C_{2}^{*},...,C_{t}^{*}$ as well as the encrypted graph intersection result ${\tilde{C}}^{*}$ for query $q_{i}\in \left\{q_{1},q_{2},...,q_{m}\right\}$. If for any probability polynomial time adversary A, it cannot differentiate the two experiments ${\mathrm{Real}}_{A}\left(\lambda \right)$ and ${\mathrm{Ideal}}_{A,S}\left(\lambda \right)$, then our scheme is considered to be secure.

Simulating the encryption. Given $q_{i}=\left(G_{1}^{i},G_{2}^{i},...,G_{t}^{i}\right)$ and leakage functions $L_{1}$ and $L_{2}$, S first checks if $q_{i}$ has been previously encountered; if it has, S provides the previous results. Otherwise, S behaves as follows: it generates *t* graphs whose scales and vertices relationships satisfy the conditions in leakage functions $L_{1}\left(q\right)=\left(Num_{1},Num_{2}\right)$ and $L_{2}=\left(L_{QP}\left(q\right),L_{IP}\left(q\right)\right)$. Then, S encrypts the *t* graphs using the hash function and proxy re-encryption algorithm to obtain the encrypted form of the *t* graphs, represented as $C_{1}^{*},C_{2}^{*},...,C_{t}^{*}$, as well as *t* hash sets $H_{1}^{*},H_{2}^{*},...,H_{t}^{*}$.

Simulating the graph intersection computation. Given $C_{1}^{*},C_{2}^{*},...,C_{t}^{*},H_{1}^{*},H_{2}^{*},...,H_{t}^{*}$, S first obtains the hash sets intersection $H_{I}^{*}$ and the encrypted vertices set intersection $C_{I}^{*}$. Then, it generates the re-encryption key $R_{TA\to DU}^{*}$ with a randomly chosen $sk^{*}$, and re-encrypts $C_{I}^{*}$. Finally, S constructs the submatrices from $C_{1}^{*},C_{2}^{*},...,C_{t}^{*}$, re-encrypts them using $R_{TA\to DU}^{*}$, and multiplies the re-encrypted submatrices to obtain the encrypted intersection matrix ${\tilde{C}}^{*}$.

Since the hash function *H* and proxy re-encryption algorithm PRE are secure, any PPT adversary A cannot distinguish the fake encrypted graphs $C_{1}^{*},C_{2}^{*},...,C_{t}^{*}$, the fake hash sets $H_{1}^{*},H_{2}^{*},...,H_{t}^{*}$, and fake encrypted intersection matrix ${\tilde{C}}^{*}$ from real ones, i.e., A cannot distinguish between experiments in the ideal world and those in the real world. Thus, we have

(6) $$\left|Pr\left[{\mathrm{Real}}_{A}\left(\lambda \right)=1\right]-Pr\left[{\mathrm{Ideal}}_{A,S}\left(\lambda \right)=1\right]\right|\leq negl\left(\lambda \right)$$

where negl is a negligible function.

Therefore, our scheme is $\left(L_{1},L_{2}\right)$-secure against an adaptive chosen query attack. □

## 7. Performance Analysis

In Table 2, we evaluate our scheme in comparison with related works across several dimensions including cryptographic primitives, privacy, cloud-assisted computation, multi-owners and multi-users. Scheme [16] computes the graph intersection of two parties. In scheme [17], multiple parties are able to collaboratively calculate the intersection of their graphs. Instead of outsourcing their graphs to a cloud server, they perform secure multi-party computation directly among the participants. Zuo et al. [15] enabled the cloud server to compute the graph intersection of multiple data owners for a single user. However, none of them support multi-users to query for graph intersection with cloud assisted computations.

### 7.1. Theoretical Analysis

In Table 3, we show the computational complexity of each phase including $KeyGen_{DU}$, $KeyGen_{TA}$, ReKeyGen, Enc, GraphIntersection, and Dec. The complexity analysis is denoted by the following operations: the exponentiation *E* in G, the exponentiation $E_{T}$ in $G_{T}$, the bilinear pairing *e*, and multiplication in $G_{T}$. We consider *t* data owners; the intersection graph of them has *s* nodes. *n* indicates that the graph encrypted has *n* nodes. $\left|G\right|$ is the size of a group element in G, and $\left|G_{T}\right|$ is the size of a group element in $G_{T}$.

In the phase of KeyGen and ReKeyGen, it needs one exponentiation in G; the key size is 1 group element in G. In the Enc phase, to encrypt a graph with *n* nodes, we need to compute *n* hashes. For each of the *n* nodes and $n^{2}$ elements in the matrix, we need to compute one exponentiation in G, one exponentiation in $G_{T}$, one pairing *e*, and one multiplication *M* in $G_{T}$, resulting in $n\cdot H+\left(n^{2}+n\right)\left(E+e+E_{T}+M\right)$ operations. In the GraphIntersection phase, the vertex set intersection and the submatrices totally contain $s+t\cdot s^{2}$ elements, and each element re-encryption requires 2E+e operations. Combining with $\left(t-1\right)\cdot s^{2}\cdot M$ operations during the multiplication of matrices, totally $\left(s+t\cdot s^{2}\right)\cdot \left(2E+e\right)+\left(t-1\right)\cdot s^{2}\cdot M$ is needed. During the Dec phase, a data user needs to decrypt the set of node intersection of size *s* and the matrix of size s×s. Each of these elements requires $\left(M+E_{T}\right)$ to decrypt, so the complexity is $\left(s+s^{2}\right)\left(M+E_{T}\right)$.

### 7.2. Experiments

In this section, we analyze the performance of our scheme through a series of experiments.

#### 7.2.1. Experimental Setting

We perform the experiments on an Ubuntu 22.04 operating system in the VMware Workstation on a PC with an i9-13900H CPU and 16 GB RAM. We implement the scheme using Go programming language based on the PBC library for Go [37]. We adopt the type A pairing which generates a pairing on the curve $y^{2}=x^{3}+x$ over the field $F_{q}$. In our experiment setting, the large prime *q* is 512 bits, and the group order of G is set to 160 bits. We instantiate hash function *H* with SHA-256. Table 4 shows the execution time of basic operations using for 100 times. We evaluate the performance using real-world graph data LastFM Asia social network [38]. The LastFM Asia social network is an undirected graph with 7627 nodes and 27,806 edges. We randomly choose subgraphs as graph data for data owners while controlling the number of common vertices among them to ensure the intersections are not empty.

#### 7.2.2. Experimental Results

**Figure 3:**
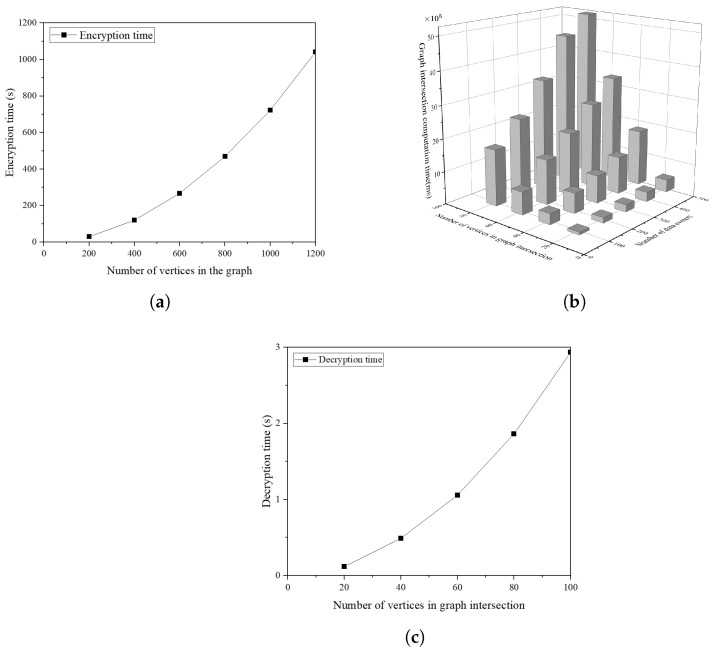
Operation time of the algorithms in our scheme. (**a**) Encryption time of data owner. (**b**) Graph intersection computation time of cloud server. (**c**) Decryption time of data user.

We demonstrate the experimental results in Figure 3.

Figure 3a shows the encryption time for graphs of different size at the data owner. Since we perform the encryption for each node and each element in the adjacency matrix, the encryption time grows with the size of the graph. As we can see, with the vertex count of the graph varying from 200 to 1200, the encryption time increases from 29.62 s to 1040.72 s. Since each data owner is required to encrypt their graph only once, the encryption time is acceptable in practice.

In Figure 3b, we show the computational cost at the cloud server. The computation time is influenced by the amount of data owners and the graph intersection size. As shown in the theoretical analysis, the cloud server needs to perform, in total, $\left(s+t\cdot s^{2}\right)\cdot \left(2E+e\right)+\left(t-1\right)\cdot s^{2}\cdot M$ operations with *t* data owners and *s* vertices in the graph intersection. We simulate this phase with data owners ranging from 100 to 500 and the vertex count in the graph intersection ranging between 20 and 80. Simulations show that the workload of the cloud server is heavy such that the computation cost rises as both the amount of the data owners and the graph intersection size increase, for example, it takes 5,143,852 s to compute the graph intersection of 500 data users with 80 common vertices. Specifically, the size of the graph intersection plays a more dominant role in determining the computation time compared to the amount of data owners.

As depicted in Figure 3c, the decryption of the data user is time saving. The decryption time is related to the size of graph intersection, and the data user only needs to perform less-time-consuming operations including exponents and multiplications in $G_{T}$. As we can see, the time of decrypting graph intersection with 20 vertices is 115.35 ms, and that with 100 vertices is 2.93 s.

## 8. Conclusions

In this paper, we introduce a privacy-preserving multi-user graph intersection scheme in the cloud-assisted IoT environment, realizing the privacy-preserving graph intersection computation. It supports multiple data users to query for the intersection of graphs of multiple data owners. We prove our scheme is secure under reasonable assumptions. The performance assessment and experimental validation on real-world graph data confirm the efficiency and practicality of our scheme. In the future, we will explore how to realize the privacy-preserving graph intersection query against malicious cloud servers and consider further improving the efficiency to make the scheme applicable to larger-scale graph data.

## Figures and Tables

**Table 1:** Summary of notations.

Notations	Description
P	public parameters
$\left(pk_{DU},sk_{DU}\right)$	public–private key pair of data user DU
(pk,sk)	public–private key pair of the trusted authority TA
$R_{TA\to DU}$	re-encryption key from trusted authority TA to data user DU
$G_{i}=\left(V_{i},E_{i}\right)$	graph of data owner $DO_{i}$
$n_{i}$	the amount of vertices in $G_{i}$
$V_{i}$	vertex set of $G_{i}$
$H_{i}$	hashed vertex set of $G_{i}$
$E_{i}$	adjacency matrix of $G_{i}$
$v_{j}^{i}$	the vertex in $V_{i}$
$e_{kl}^{i}$	the element in adjacency matrix $E_{i}$
$C^{i}=\left(C_{v}^{i},C_{e}^{i}\right)$	the encrypted form of $G_{i}$
$\tilde{C}=\left({\tilde{C}}_{I},{\tilde{C}}_{e}\right)$	the encrypted graph intersection

**Table 2:** Comparison of functionalities with existing schemes.

Schemes	Cryptographic Primitives	Privacy	Cloud-Assisted Computation	Multi-Owners	Multi-Users
[16]	Paillier encryption	*√*	×	×	×
[17]	Lifted-ElGamal threshold encryption	*√*	×	*√*	*√*
[15]	ElGamal encryption	*√*	*√*	*√*	×
Our scheme	Proxy re-encryption	*√*	*√*	*√*	*√*

**Table 3:** Theoretical analysis of our scheme.

Algorithms	Computational Cost	Output Size
$KeyGen_{DU}$	1E	$1\left|G\right|$
$KeyGen_{TA}$	1E	$1\left|G\right|$
ReKeyGen	1E	$1\left|G\right|$
Enc(one data owner)	$n\cdot H+\left(n^{2}+n\right)\left(E+e+E_{T}+M\right)$	$\left(n^{2}+n\right)(\left|G_{T}\right|+\left|G\right|)+n\cdot \left|H\right|$
GraphIntersection	$\left(s+t\cdot s^{2}\right)\cdot \left(2E+e\right)+t\cdot s^{2}\cdot M$	$\left(s^{2}+s\right)\left(\left|G_{T}\right|+2\left|G\right|\right)$
Dec	$\left(s+s^{2}\right)\left(M+E_{T}\right)$	-

**Table 4:** Execution time of basic operations.

Operation (×100)	Exponent in G	Exponent in $G_{T}$	Multiplication in $G_{T}$	Bilinear Paring	*H*
time	62.70 ms	5.30 ms	160.08 µs	41.58 ms	138.61 µs

## Data Availability

Data are contained within the article.
